# P03-022 - Calprotec in chronic nonbacterial osteomyelitis

**DOI:** 10.1186/1546-0096-11-S1-A220

**Published:** 2013-11-08

**Authors:** J Brunner

**Affiliations:** 1 Pediatrics, Medical University Innsbruck, Innsbruck, Austria

## Introduction

The cytoplasmic S100 proteins derived from cells of myeloid origin. Calprotectin (MRP8/14 protein complex) might be a biomarker either for autoinflammation and autoimmunopathy. Since autoinflammatory diseases might be a diagnostic challenge calprotectin may be helpful in the diagnosis of autoinflammatory diseases. Chronic nonbacterial osteomyelitis (CNO) is an autoinflammatory, noninfectious disease. CNO describes a wide spectrum from a monofocal bone lesion to the chronic recurring multifocal osteomyelitis (CRMO). Laboratory and histopathological findings are nonspecific. In some patients systemic inflammatory signs such as elevated acute phase proteins cannot be found.

## Case Report

To test the ability of Calprotectin (MRP8/14 protein complex) serum concentrations to monitor disease activity in patients with CNO.

## Methods

Serum concentrations of Calprotectin (MRP8/14 protein complex) in a patient with CNO were determined by a sandwich ELISA.

## Results

Calprotectin (MRP8/14) level were raised heralding active disease when acute phase proteins (CrP, erythrocyte sedimentation rate). The calprotectin level was 7872,7 ng/ml (normal range 0-3000 ng/ml).

## Discussion

Calprotectin (MRP8/14) serum concentrations correlate closely with disease activity and may herald a flare before clinical manifestation. Therefore MRP8/14 serum concentrations are a biomarker indicating disease activity in CNO patients.

## Competing interests

None Declared.
